# 

**Published:** 1873-08

**Authors:** 


### CONTENTS:

### ORIGINAL COMMUNICATIONS.

ARTICLE I.—California State Dental Association........................................ 1[illegible]

ARTICLE II.—Nitrous Oxide Experiments. By. S. P. Cutler, M. D., D. D. S................ 15

ARTICLE III.—Porcelain Impression Cups for Taking Impressions of the Teeth. By B. M. Wilkerson, M. D., D. D. S................................................ 1[illegible]

### MONTHLY SUMMARY.

On the Use of Artificial Respiration and Transfusion as a means of Preserving Life......... 15

A New Method for Healing Ulcers................................................ 15

Neuralgia and Sciatica Cured by Turpentine..................................... 15[illegible]

The Physiology of the Nervous System........................................... 16[illegible]

Fatal Poisoning from Carbolic Acid............................................. 16[illegible]

